# Relationship Between Foot Length and Gestational Age in Pakistan

**DOI:** 10.1177/2333794X20974206

**Published:** 2020-11-20

**Authors:** Shiyam Sunder Tikmani, Sana Roujani, Syed Iqbal Azam, Haleema Yasmin, Khadija Bano, Saleem Jessani, Sayyeda Reza, Elizabeth M. McClure, Robert L. Goldenberg, Sarah Saleem

**Affiliations:** 1 The Aga Khan University, Karachi, Pakistan; 2 Jinnah Postgraduate Medical Center, Karachi, Sindh, Pakistan; 3 Research Triangle Institute International, Durham, NC, USA; 4 Columbia University, New York, NY, USA

**Keywords:** gestational age, last menstrual period, Ballard scoring, foot length

## Abstract

Preterm births have a high risk of mortality. Therefore, knowledge of the gestational age (GA) at birth is crucial to guide the appropriate management of a newborn. Common methods for estimating GA such as the last menstrual period, ultrasonography, and post-natal Ballard scoring have some limitations. This study aimed to determine the relationship between foot length and GA to develop and validate an equation for predicting GA of Pakistani newborns. We conducted a prospective study in a large obstetric hospital in Pakistan. Data for this analysis were extracted from the hospital files of eligible women by trained study midwives. Midwives were also trained in performing the Ballard examination and taking foot length using a disposable measuring tape within an hour of the birth. The GA was calculated using an android-based GA calculator. Simple and multiple linear regression were used to construct predicting equations for GA. Both the foot length and GA were available for 1542 cases. The median GA was 34.5 (IQR 4.7) weeks and the median foot length was 7 cm (IQR 1.4). There was a positive linear relationship between foot length and GA (*r*^2^ 81.7%, *P*-value < .001). Stratified analysis showed an *r*^2^ of 81.7% for males and 81.6% for females. The *r*^2^ for stillbirths was 84.1% and, 82.3% for live births. The *r*^2^ for macerated stillbirths was 88.6% and 90.6% for fresh stillbirths. In resource poor settings, the use of foot length can estimate GA in both live births and stillbirths and can easily identify preterm infants.

## Background

Pakistan has the highest neonatal mortality across the globe with a neonatal mortality rate (NMR) of 43/1000 live births (LB).^1,2^ In Pakistan, 37% of deliveries occur at home and 42% of deliveries occurred at home in the rural areas of Sindh province alone.^2^ Most of these home deliveries are attended by unskilled birth attendants.^2^ According to the recent Pakistan Demographic and Health Survey (PDHS-2018), the most common causes of neonatal deaths are infections and prematurity,^3^ and the majority of neonatal deaths occur in preterm newborns.

Knowledge about the gestational age (GA) at birth is crucial for the appropriate management to reduce risks for newborns, especially in preterm births.^4^ There are different methods of estimating GA. During the antenatal period, GA is estimated using the first day of the last menstrual period (LMP) and ultrasonography.^5^ LMP is a common method to assess GA if the menstrual cycle is regular. However, in the case of an irregular cycle, LMP may not be an accurate method to estimate the GA. Ultrasound (US) examination is considered to be a gold standard for dating^5^; however, dating using US is considered accurate when it is carried out in the first trimester with a variation of ±10 days and is often unavailable in resource-poor regions.^6^

If the antenatal GA is unknown, then the post-natal assessment may provide a reasonable estimation of GA. Postnatal assessment of GA can be assessed by using different techniques, for example, through Dubowitz scoring,^7^ New Ballard Scoring,^8^ Eregie model,^9^ and measurement of different parts of the body, such as chest circumference, leg length, foot length, etc.^10,11^ Nevertheless, both antenatal and postnatal estimations of GA have some limitations.

In low-middle-income countries like Pakistan where female illiteracy is high, women generally are unable to recall their LMP^10^ and a good quality first-trimester ultrasound report is not commonly available. In such cases, Ballard scoring is the most commonly used post-natal method to determine the GA of the baby. Ballard scoring consists of 2 components: (1) physical and (2) neuromuscular maturity assessment. However, Ballard scoring is technical and subjective for health care workers to use and estimate the GA accurately.^10^ Health care workers can measure foot length with minimal training and can estimate GA.

The Global Action Report on Preterm Birth, “Born too soon” emphasized using simple approaches of estimating GA for early identification of preterm infants and their management.^12^ Recently, a study from Ethiopia reported that foot length could be used as a diagnostic tool for predicting LBW and prematurity.^10^ However, in Pakistan, there is no standardized foot length chart available to assess GA. Therefore, this study aimed to determine the relationship between GA and foot length to construct a simple regression equation for GA assessment for Pakistani newborns and to validate this equation with other existing equations to estimate GA.

## Methods

### Study Setting, Design, and Population

Data for this analysis were extracted from a large prospective, observational cohort study carried out in Pakistan with the primary objective to determine the cause of death in stillbirth and preterm live-born infants.^13^

The primary study was conducted in the Department of Obstetrics and Gynecology, Jinnah Postgraduate Medical Center (JPMC), Karachi, Pakistan. RTI International (RTI) served as the data center and provided data management and analytic support. The Department of Obstetrics and Gynecology has 135 beds and around 15 000 annual deliveries with approximately 15% preterm births and 3.5% stillbirths. The data for the study were collected from September 3rd, 2018 to December 31st 2019.

Pregnant women ≥18 years of age admitted with an imminent preterm delivery based on clinical indications (20-36, 6 weeks gestation) or women with a known stillbirth (intrauterine fetal death ≥20 weeks) were included in the study. Induced abortions (<20 weeks), live births with unknown GA, lethal congenital malformations, neonates with limb deformities, and missing foot length measurements were excluded.

### Ethical Approvals

The primary study was approved by the ethical review committees of Aga Khan University (5212-CHS-ERC-18), Jinnah Post-Graduate Medical Center (2018-GENL/8740), National Institute of Child Health (IERB-11/2018), and National Bioethics Committee of Pakistan (NBC-312/18/RDC/3816). Written informed consent was taken from each participant of the study.

### Data Collection Procedure

GA was calculated through an Android-based GA calculator with a predefined algorithm using the hierarchy of methods established by the American College of Obstetrics and Gynecology,^14^ using the report of reliable LMP, ultrasound examination, and Ballard examination. The LMP and ultrasound data were extracted from the woman’s hospital file. The trained study midwives carried out Ballard examinations. All data were then entered into the GA calculator.^14^ Based on this information, the GA calculator then provided the best GA estimate.

The foot length of the right foot was measured by trained midwives using a disposable measuring tape within an hour of birth from the mid-point of the heel to the end of the longest toe. Among the live births, to minimize the effect of the plantar grasp reflex on the length of the foot, midwives were trained to hold the ankle with a finger placed on the dorsum of the foot to keep the foot straight. An average of 3 readings of foot length in centimeters was used for the final analysis.

Study midwives were trained for data extraction from the women’s hospital files, carrying out a Ballard examination, and measuring the foot length of the baby. They were also trained in operating the GA calculators. Random checks were carried out by the study supervisor nurse to verify the measurement of the foot length and appropriateness of the use of the GA calculator by the midwives.

### Statistical Analysis

Data were analyzed using SPSS version 19 (SPSS Inc., Chicago, IL, USA). The normality of GA and foot length was checked through the Kolmogorov-Smirnov test. For GA and foot length, median and interquartile ranges (IQR) are reported. Frequencies and percentages were calculated for categorical variables. For comparison of characteristics of the participants, Student *t*-tests for continuous variables and chi-square tests for categorical variables were used.

A Scatter plot was made to assess the linearity between GA and foot length. Co-efficient of determination (*r*^2^) and correlation (*r*) were calculated. Separate scatter plots were made to assess linearity by sex, livebirth, stillbirth, and presence of maceration. Linear regression modeling was done for estimating predictive values of foot length for GA. For the assumption of the normality of residuals, a normality plot of residuals and predicted GA was constructed using PP plots and assessing heteroscedasticity. Cook’s distance and Df Beta were estimated to assess influential values. Multiple linear regression equation was made which is adjusted for sex and birth status.

Cross-validation of the model was done by randomly dividing the data set into 2 subsamples (1/3 and 2/3). The statistical model was generated with 2/3rd of the data. The model was then used to predict GA in the 1/3rd subsample. The standardized error term was calculated based on the predicted GA using the formula: (observed GA − predicted GA)^2^/(predicted GA).

For validation, the foot length of Pakistani neonates for estimating GA was assessed using regression equations reported by different studies.^15-20^ The mean difference between observed and predicted GA was compared using a paired t-test. The mean differences and 95% CI were plotted in a graph.

## Results

A total of 20 169 women delivered during the study period. Of these births, 1066 (6.6%) were stillbirths and 1844 (9.14%) were preterm live births. A total of 2696 women were screened for imminent preterm birth or stillbirth and 2905 babies were born. Out of 2548 eligible mother-infant pairs, 2044 provided consent and were enrolled in this study. Of these, 607 were stillbirths and 1437 were preterm live births. GA was available for 1436 preterm neonates and 554 stillbirths. Both foot length and GA were available for 1542 cases that were analyzed to determine this relationship, 767 (49.7%) were male, 773 (50.1%) were female, and in 2 (0.1%) cases, information on sex was not known. Of 1542 infants, 1132 (73.4%) were live births and 410 (26.6%) were stillbirths (Table 1).

**Table 1.** Characteristics of Infants.

Characteristics	Livebirth (n = 1130)	Stillbirth (n = 410)	Total (n = 1542)
Gender			
Male	560 (49.6%)	207 (50.5%)	767 (49.8%)
Female	570 (50.4%)	203 (49.5%)	773 (50.2%)
Gestational age (weeks) *Median (IQR)*	34.6 (3.9)	33.3 (7.4)	34.5 (4.7)
Preterm	904 (80.0%)	316 (77.1%)	1220 (79.1%)
Foot length (cm) *Median (IQR)*	7.1 (1.2)	6.5 (1.9)	7.0 (1.4)

The Shapiro-Wilk test (*P* ≥ .05) and a visual inspection of histograms of maternal age, GA and foot length, showed that these variables were not normally distributed. The median maternal age was 26 (IQR 7) years, the median GA was 34.5 (IQR 4.7) weeks; median foot length was 7 (IQR 1.4) cm (Table 1).

**Figure 1.**
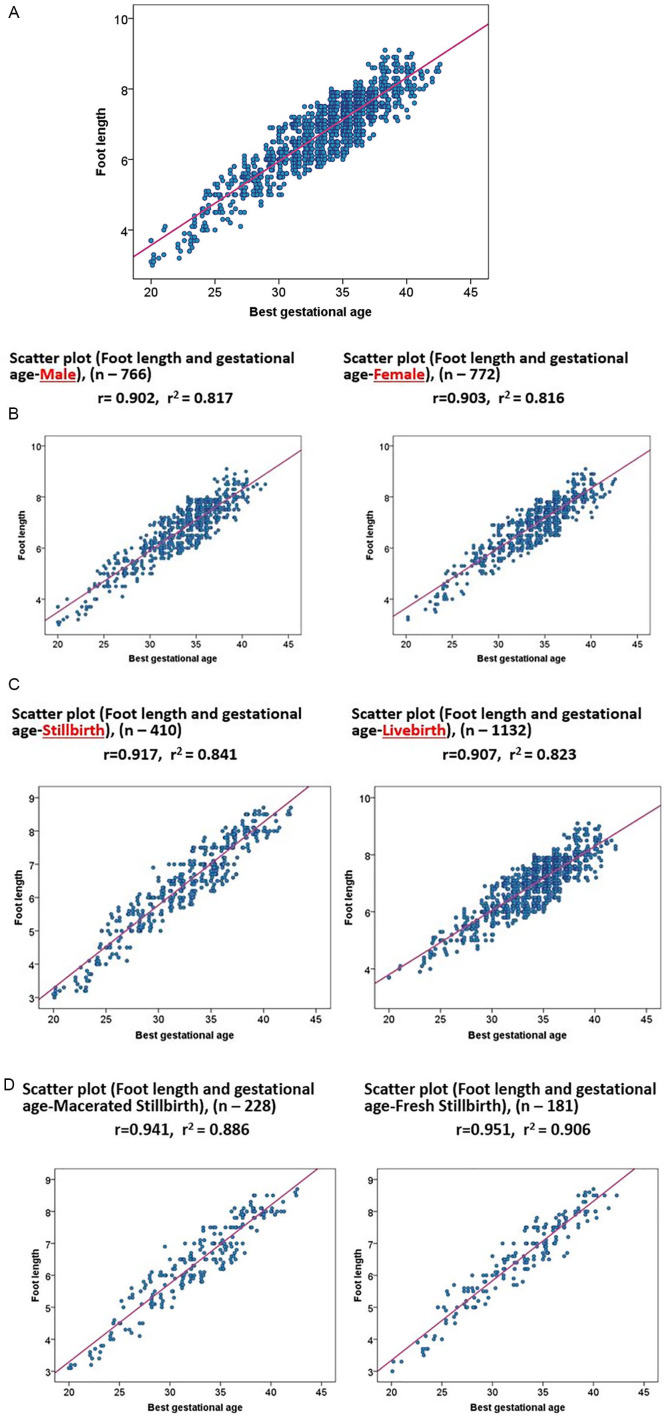
Scatter plot showing a linear relationship between foot length and gestational age (A-D). (A) Overall relationship between gestational age and foot length with correlation coefficient of 0.903 and coefficient of determination 0.817 (n = 1542). (B) Relationship between foot length and gestational age by sex. (C) Relationship of foot length and gestational age by livebirth and stillbirth. (D) Relationship between foot length and gestational age by presence and absence of maceration among stillbirth.

**Figure 2.**
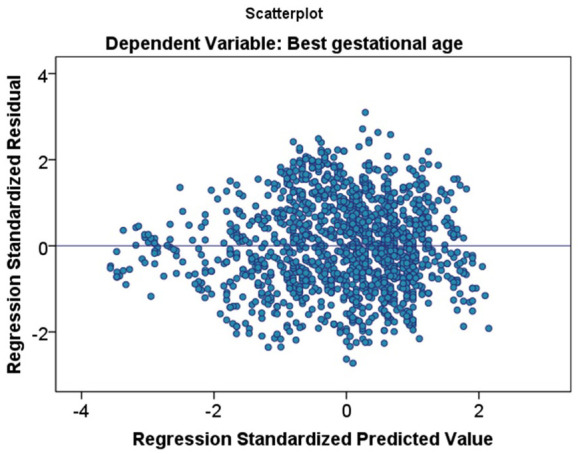
Graph showing distribution regression standardized residuals.

Figure 1 shows the scatter plots of the relationship between foot length and GA. There is a positive linear relationship between foot length and GA (*r*^2^ 81.7%, *P*-value < .001). Standardized errors are provided in Figure 2, showing near-normal distribution. Stratified analysis showed an *r*^2^ 81.7% for males and 81.6% for females. For stillbirths, the *r*^2^ was 84.1% and for livebirths, the *r*^2^ was 82.3%. Stratification by the presence of maceration resulted in an *r*^2^ of 90.6% for fresh stillbirths and an *r*^2^ of 88.6% for macerated stillbirths (Figure 1).

**Figure 3.**
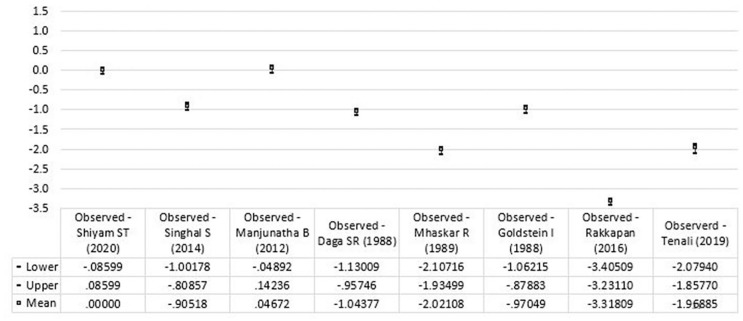
Mean difference and 95% CI of observed and predicted values using different prediction equations.

The mean difference of observed and predicted GA with CIs, using different equations are shown in Figure 3. The difference of observed and predicted GA of this study is consistent with the difference in the observed value of this study and predicted value of the equation of Manjunatha et al.^21^ However, all other equations (Table 2) appear to underestimate the GA compared to mean difference of the observed and predicted value of this study (Figure 3).

**Table 2.** Simple Linear Regression Equations Constructed by Different Studies.

Author	Regression equation	*R*-Square
Tenali and Tenali^16^	GA = 21.84 + (2.09*FL)	0.9
Rakkappan and Kuppusamy^17^	GA = 15.343 + (3.183*FL)	0.65
Singhal et al^18^	GA = 6.278 + (4.15*FL)	0.86
Manjunatha et al^21^	GA = 5.60 + (4.11*FL)	0.97
Mhaskar et al^19^	GA = 12.79 + (3.36*FL)	0.85
Daga et al^20^	GA = 11.55 + (3.41*FL)	0.84

Abbreviations: FL, foot length; GA, gestational age.

## Discussion

In this study, a positive linear relationship was observed between GA and foot length (*r*^2^ = 81.7%). Streeter in 1920, first observed that the foot length co be used as a proxy for estimation of GA.^15^ Many studies since then have shown a positive linear relationship between GA and foot length.^16-20,22^ However, the coefficient of determination (*r*^2^) of foot length and GA is different in different studies. Singhal et al found a positive relationship between foot length and GA with an *r*^2^ of 93%.^18^ Mhaskar et al reported that foot length correlated very well with the GA with an *r*^2^ of 85%.^19^ Rakkappan and Kuppusamy reported a lower *r*^2^ (65%) between foot length and GA.^17^ In this study, the correlation between foot length and GA was 82.3% in live births and 84.1% in stillbirths, 88.6% macerated stillbirths, and 90.6% for fresh stillbirths.

There are wide variations in the methods used across the studies. These variations include sample size, the GA of infants included in the analysis, method of measurement of GA, different tools to measure foot length, and different units used for the foot length measurement. However, most of the studies reported that the landmark for measuring foot length was similar to that used in this study. Several studies measured GA using different methods including LMP,^18^ US examination,^20^ clinical examination,^21^ and the Ballard score.^16^ Similarly, foot length was measured using different measuring equipment. The most commonly used equipment was a Vernier caliper,^15,16,17,19,20,21^ followed by transparent rigid scales and steel or flexible tapes.^10,18,22^ The unit of measurement was also different across studies. Some used centimeters^16-18^ and some millimeters.^20,21^ However, almost all studies measured foot length from the mid-point of the heel to the longest toe. In this study, we determined GA using an algorithm and foot length by a disposable flexible paper tape in centimeters from mid-point of the heel to the longest toe.

Due to these variations, we compared the estimated GA using foot length from this study in the regression equations constructed by different studies (Table 2).^16-21^ Most of the prediction equations underestimate the GA compared to the predicted model developed in this study except for the model by Manjunatha et al.^21^ However, the mean difference of observed and predicted GA using different equations (Table 1) shows the minimal difference with precise confidence limits.

To the best of our knowledge, this is the first study in Pakistan to determine the relationship between foot length and GA. Our study had several strengths. The data were prospectively collected. The GA was calculated using an algorithm through an Android application. The relationship of foot length and GA was compared by gender, stillbirth and live birth, macerated, and fresh stillbirth, which have not been reported in any single study so far. Foot length was measured by well-trained midwives with standardization methods. We excluded infants with congenital malformations and limb deformities from this study as this might have affected the GA estimation by 2 to3 weeks.^22^

However, there were some limitations to the study. A flexible disposable measuring tape was used to measure the foot length. However, we believe that standardization exercises and taking an average of 3 readings minimized the error. We also did not perform in-depth intra- or inter-observer variation analysis; however, the study midwives were trained with quarterly refresher trainings and were monitored by a senior study nurse, thus minimizing the inter-observer variation. In conclusion, a simple measurement of foot length appears to be method to effectively estimate GA. Having this additional tool can help identify preterm infants in low resource settings. Ultimately, better assessment of GA can help providers to initiate treatment and refer sick babies for specialized care leading to reduction of mortality in preterm neonates in a resource-limited setting.
